# Crystal structures and Hirshfeld surface analyses of 4-benzyl-6-phenyl-4,5-di­hydro­pyridazin-3(2*H*)-one and methyl 2-[5-(2,6-di­chloro­benz­yl)-6-oxo-3-phenyl-1,4,5,6-tetra­hydropyridazin-1-yl]acetate

**DOI:** 10.1107/S2056989019013707

**Published:** 2019-10-22

**Authors:** Said Dadou, Sevgi Kansiz, Said Daoui, Fouad El Kalai, Cemile Baydere, Rafik Saddik, Khalid Karrouchi, Necmi Dege, Noureddine Benchat

**Affiliations:** a Laboratory of Applied Chemistry and Environment (LCAE), Faculty of Sciences, Mohamed I University, 60000 Oujda, Morocco; b Department of Physics, Faculty of Arts and Sciences, Ondokuz Mayıs University, 55139 Kurupelit, Samsun, Turkey; c Laboratory of Organic Synthesis, Extraction and Valorization, Faculty of Sciences, Ain Chok, University Hassan II, Casablanca, Rabat, Morocco; d Laboratory of Plant Chemistry, Organic and Bioorganic Synthesis, URAC23, Faculty of Science, B.P. 1014, GEOPAC Research Center, Mohammed V University, Rabat, Morocco

**Keywords:** crystal structure, pyridazine, hydrogen bonding, Hirshfeld surface analysis

## Abstract

The asymmetric units of the title compounds both contain one nonplanar mol­ecule. In 4-benzyl-6-phenyl-4,5-di­hydro­pyridazin-3(2*H*)-one, C_17_H_14_N_2_O, (**I**), the phenyl and pyridazine rings are twisted with respect to each other, making a dihedral angle of 46.69 (9)°; the phenyl ring of the benzyl group is nearly perpendicular to the plane of the pyridazine ring, the dihedral angle being 78.31 (10)°. In methyl 2-[5-(2,6-di­chloro­benz­yl)-6-oxo-3-phenyl-1,4,5,6-tetra­hydropyridazin-1-yl]acetate, C_20_H_16_Cl_2_N_2_O_3_, (**II**), the phenyl and pyridazine rings are twisted with respect to each other, making a dihedral angle of 21.76 (18)°, whereas the phenyl ring of the di­chloro­benzyl group is inclined to the pyridazine ring by 79.61 (19)°. In the crystal structure of (**I**), pairs of N—H⋯O hydrogen bonds link the mol­ecules into inversion dimers with an *R*_2_^2^(8) ring motif. In the crystal structure of (**II**), C—H⋯O hydrogen bonds generate dimers with *R*_1_^2^(7), *R*_2_^2^(16) and *R*_2_^2^(18) ring motifs. The Hirshfeld surface analyses of compound (**I**) suggests that the most significant contributions to the crystal packing are by H⋯H (48.2%), C⋯H/H⋯C (29.9%) and O⋯H/H⋯O (8.9%) contacts. For compound (**II**), H⋯H (34.4%), C⋯H/H⋯C (21.3%) and O⋯H/H⋯O (16.5%) inter­actions are the most important contributions.

## Abstract 2

In each asymmetric unit of the title compounds, one independent mol­ecule is present. In the crystal structure of 4-benzyl-6-phenyl-4,5-di­hydro­pyridazin-3(2*H*)-one, adjacent mol­ecules are linked by a pair of N—H⋯O hydrogen bonds, forming inversion dimers with an 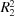
(8) ring motif. The crystal structure of methyl 2-[5-(2,6-di­chloro­benz­yl)-6-oxo-3-phenyl-1,6-di­hydro-pyridazin-1-yl]acetate displays inter­molecular C—H⋯O inter­actions.

## Chemical context   

Pyridazines are an important family of six-membered aromatic heterocycles (Akhtar *et al.*, 2016 ▸). The chemistry of pyridazinones has been an inter­esting field of research for decades and this nitro­gen-containing heterocycle has become a scaffold of choice for the development of potential drug candidates (Dubey & Bhosle, 2015 ▸). Pyridazinone is an important pharmacophore possessing a wide range of biological applications (Asif, 2014 ▸). A review of the literature revealed that substituted pyridazinones have received a lot of attention in recent years because of their significant potential as anti­microbial (Sönmez *et al.*, 2006 ▸), anti­hypertensive (Siddiqui *et al.*, 2011 ▸), anti­depressant (Boukharsa *et al.*, 2016 ▸), anti-HIV (Livermore *et al.*, 1993 ▸) and anti-inflammatory (Barberot *et al.*, 2018 ▸) agents.

We report herein the syntheses and crystal and mol­ecular structures of the pyridazinone derivatives 4-benzyl-6-phenyl-4,5-di­hydro­pyridazin-3(2*H*)-one, (**I**), and methyl 2-[5-(2,6-di­chloro­benz­yl)-6-oxo-3-phenyl-1,4,5,6-tetra­hydropyridazin-1-yl]acetate, (**II**), as well as the analyses of their Hirshfeld surfaces.

## Structural commentary   

**Figure 1:**
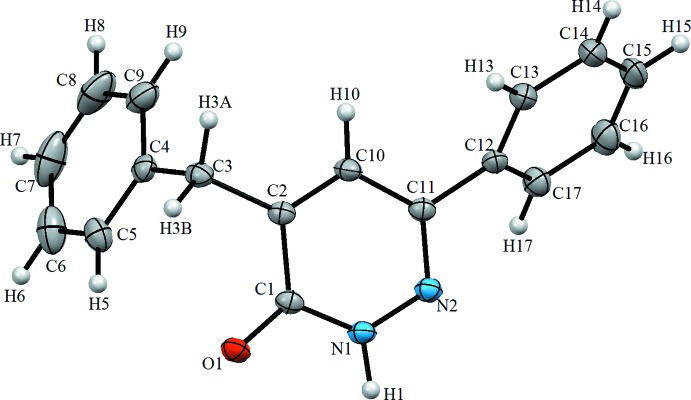
The mol­ecular structure of compound (**I**), with the atom labelling. Displacement ellipsoids are drawn at the 30% probability level.

**Figure 2:**
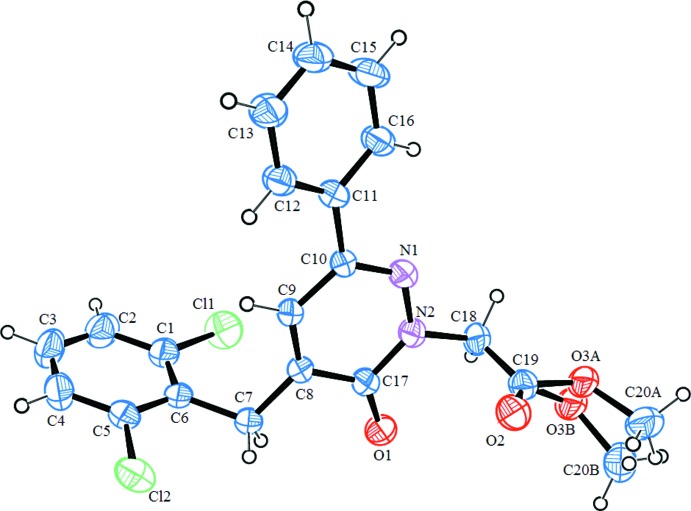
The mol­ecular structure of compound (**II**), with the atom labelling. Displacement ellipsoids are drawn at the 30% probability level.

The mol­ecular structures of compounds (**I**) and (**II**) are illustrated in Figs. 1 ▸ and 2 ▸, respectively. The common moiety for (**I**) and (**II**) is 4-benzyl-6-phenyl-4,5-di­hydro­pyridazin-3(2*H*)-one. The differences between (**I**) and (**II**) pertain to the addition of two chloro groups at the C2 and C6 ring positions of the benzyl group and N-alkyl­ation of pyridazinone at the 2-position with an ethyl acetate group for (**II**). In (**I**), the phenyl ring (atoms C12–C17) and the pyridazine ring (N1/N2/C11/C10/C2/C1) are twisted with respect to each other, making a dihedral angle of 46.69 (9)°; the phenyl ring of the benzyl group (C4–C9) is nearly perpendicular to the pyridazine ring, with a dihedral angle of 78.31 (10)° (Fig. 1 ▸). In (**II**), the phenyl ring (C11–C16) and the pyridazine ring (N1/N2/C17/C8/C9/C10) are twisted with respect to each other, making a dihedral angle of 21.76 (18)°; the phenyl ring (C1–C6) of the benzyl group is inclined to the pyridazine ring by 79.61 (19)°. The meth­oxy group in (**II**) is disordered over two sets of sites with an occupancy ratio of 0.626 (11):0.374 (11) (Fig. 2 ▸). In (**I**), the carbonyl group has a C1=O1 bond length of 1.243 (2) Å, and the C1—N1 and C11—N2 bond lengths in the pyridazine ring are 1.363 (2) and 1.304 (2) Å, respectively. The corresponding values in (**II**) are 1.229 (5) Å for C17=O1, 1.388 (5) Å for C17—N2 and 1.299 (4) Å for C10—N1. The N1—N2 bond lengths in the structures are virtually the same, with values of 1.348 (2) Å in (**I**) and 1.353 (4) Å in (**II**).
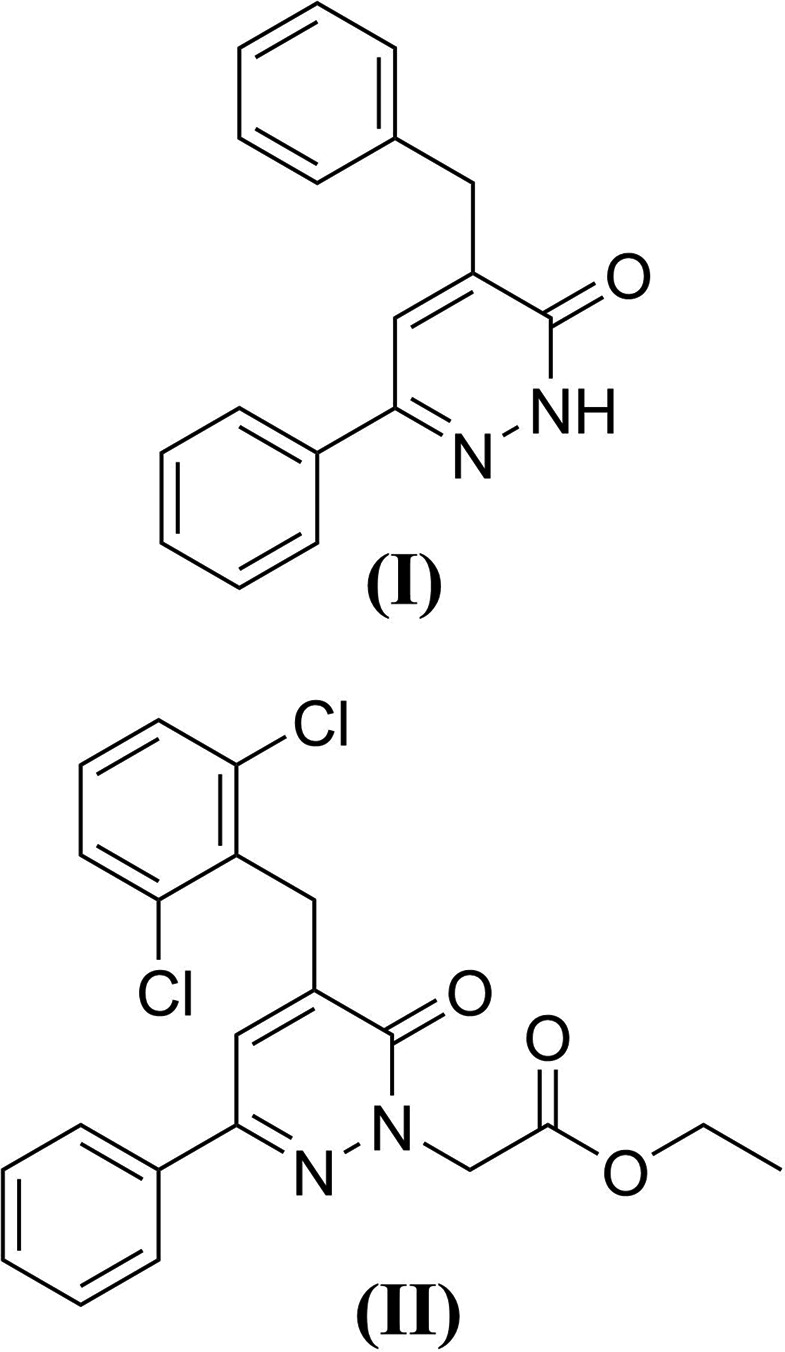


## Supra­molecular features   

**Figure 3:**
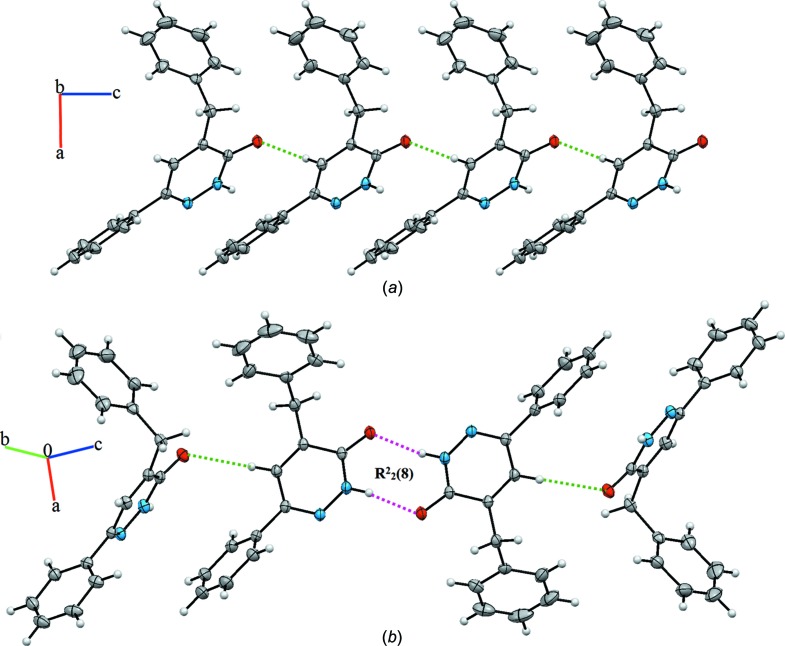
(*a*) A portion of the crystal structure of compound (**I**), viewed along the *b*-axis direction, emphasizing the C—H⋯O inter­actions, shown as green dashed lines. (*b*) A view of the crystal packing, showing additional N—H⋯O hydrogen bonds (pink dashed lines) forming inversion dimers with an 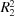
(8) ring motif (Table 1 ▸).

In the crystal of (**I**), mol­ecules are linked by C—H⋯O hydrogen bonds (Table 1 ▸) between the methine C10—H10 group and the carbonyl O1 atom of an adjacent mol­ecule (Fig. 3 ▸ *a*), and by a pair of N—H⋯O hydrogen bonds forming inversion dimers with an 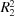
(8) ring motif (Fig. 3 ▸ *b*). The dimers are linked by weak π–π inter­actions, with a centroid-to-centroid distance of 3.957 (2) Å for *Cg*1 and *Cg*2, where *Cg*1 is the centroid of the N1/N2/C11/C10/C2/C1 ring and *Cg*2 that of the C12–C17 phenyl ring. In this way, a three-dimensional network is formed.

**Figure 4:**
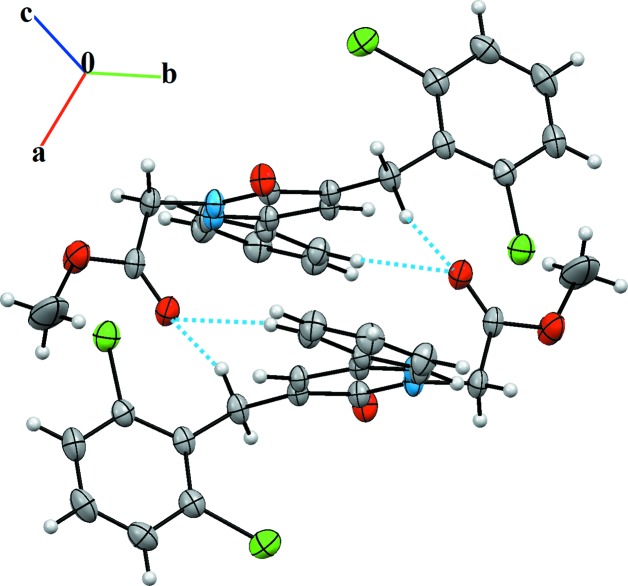
A portion of the crystal structure of compound (**II**). Dashed light-blue lines denote inter­molecular C—H⋯O hydrogen bonds.

In the crystal of (**II**), mol­ecules are connected *via* C—H⋯O hydrogen bonds between aryl groups and the carbonyl O1 atom (Table 2 ▸), whereby C9—H9⋯O2^i^ and C12—H12⋯O2^i^ hydrogen bonds generate 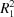
(7) motifs (Fig. 4 ▸); likewise, 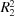
(16) and 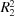
(18) ring motifs are also observed.

## Database survey   

A search of the Cambridge Structural Database (CSD, Version 5.40, update of February 2019; Groom *et al.*, 2016 ▸) for the 4-benzyl-6-phenyl­pyridazin-3(2*H*)-one skeleton yielded three hits, namely 4-(2-chloro-6-fluoro­phen­yl)-2,5-dioxo-8-phenyl-1,2,3,4,5,6-hexa­hydro­pyrido[2,3-*d*]pyridazine (BARQOA; Pita *et al.*, 2000 ▸), 4-(2-chloro-5-nitro­phen­yl)-2,5-dioxo-8-phenyl-1,2,3,4,5,6-hexa­hydro­pyrido[2,3-*d*]pyridazine (BARQUG; Pita *et al.*, 2000 ▸) and 4-benzyl-6-*p*-tolyl­pyridazin-3(2*H*)-one (YOTVIN; Oubair *et al.*, 2009 ▸). In YOTVIN, the mol­ecules are connected two-by-two through N—H⋯O hydrogen bonds, with an 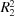
(8) graph-set motif, building up a pseudo-dimer arranged around an inversion centre. In the three structures, the C—N bonds in the pyridazine rings correspond to C1—N1 in the structure of (**I**) [1.363 (2) Å], with a value of 1.363 (2) Å for BARQOA, 1.364 (7) Å for BARQUG and 1.350 (2) Å for YOTVIN. The pyridazinone ring in each mol­ecule is essentially planar, as in the structures of (**I**) and (**II**). The conformations of all three compounds resemble those of compounds (**I**) and (**II**), with the dihedral angles between the planes of the pyridazine and phenyl rings varying in the range 27.35–82.0°, compared to 46.69 (9) and 21.76 (18)° in (**I**) and (**II**), respectively.

## Hirshfeld surface analysis   

**Figure 5:**
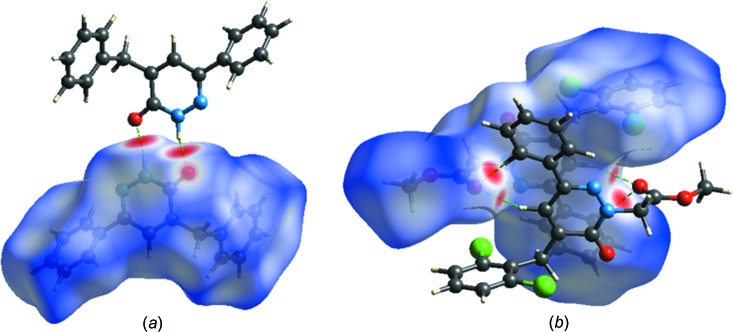
*d*_norm_ mapped on Hirshfeld surfaces for visualizing the inter­molecular inter­actions of (*a*) compound (**I**) and (*b*) compound (**II**).

Hirshfield surface analyses (Spackman & Jayatilaka, 2009 ▸) were carried out using *CrystalExplorer* (Version 17.5; Turner *et al.*, 2017 ▸). The Hirshfeld surfaces and their associated two-dimensional fingerprint plots were used to qu­antify the various inter­molecular inter­actions in the structures of the title compounds. Calculations of the mol­ecular Hirshfeld surfaces (HS) were performed using a standard (high) surface resolution with the three-dimensional *d*_norm_ surfaces mapped over a fixed colour scale of −0.6062 (red) to 1.3165 a.u. (blue) for (**I**) and of −0.2803 (red) to 1.5329 a.u. (blue) for (**II**). The red spots on the surface indicate the contacts involved in hydrogen bonding. Fig. 5 ▸(*a*) illustrates the inter­molecular N—H⋯O hydrogen bonding in (**I**), with *d*_norm_ mapped on the Hirshfeld surface. Likewise, C—H⋯O hydrogen bonding is visualized in Fig. 5 ▸(*b*) for compound (**II**).

**Figure 6:**
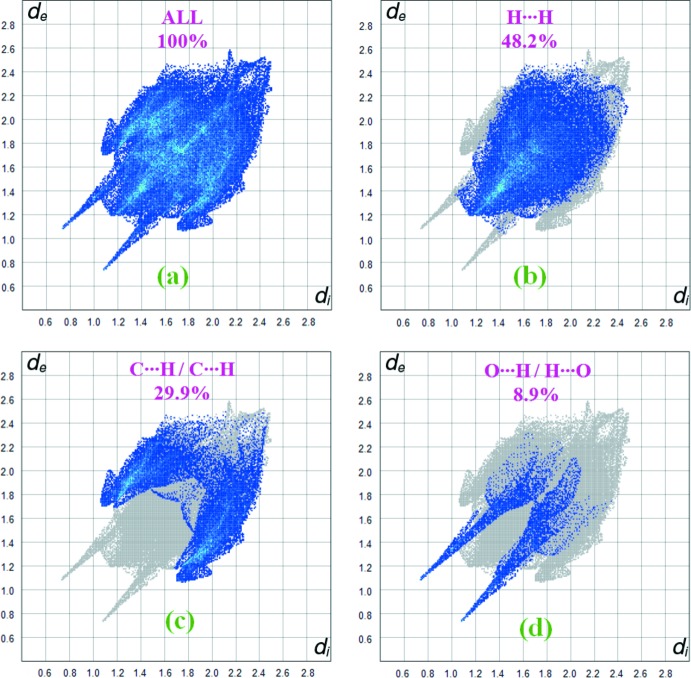
Two-dimensional fingerprint plots of compound (**I**) showing the relative contributions of the atom pairs to the Hirshfeld surface.

Fig. 6 ▸ shows the two-dimensional fingerprint plot of the sum of the contacts contributing to the Hirhsfeld surface of compound (**I**), represented in normal mode. H⋯H contacts clearly make the most significant contribution to the Hirshfeld surface (48.2%). A significant contribution of H⋯H inter­actions to the total HS (72.2%) was also reported by Ilmi *et al.* (2019 ▸) for a similar compound. In addition, C⋯H/H⋯C and O⋯H/H⋯O contacts contribute 29.9 and 8.9%, respectively, to the Hirshfeld surface. In particular, the O⋯H/H⋯O contacts indicate the presence of inter­molecular N—H⋯O and C—H⋯O inter­actions.

**Figure 7:**
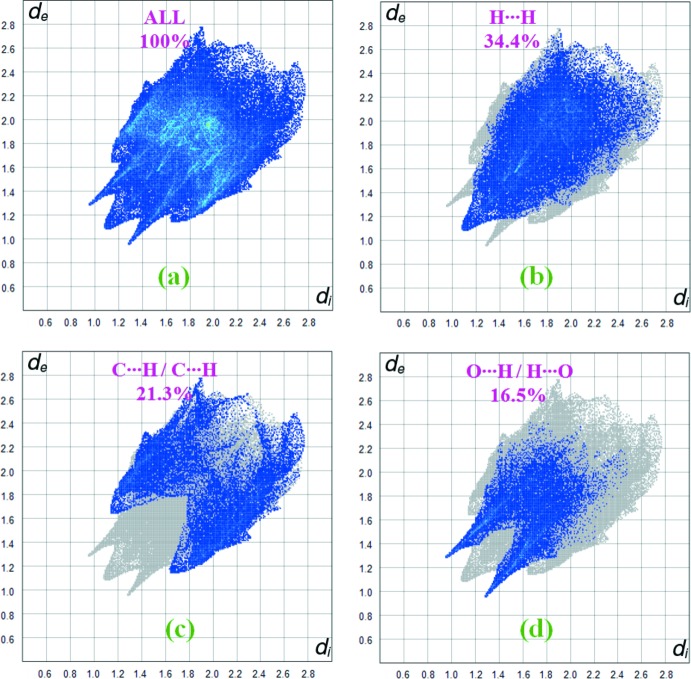
Two-dimensional fingerprint plots of compound (**II**) showing the relative contributions of the atom pairs to the Hirshfeld surface.

Similarly, Fig. 7 ▸ illustrates the two-dimensional fingerprint plot of the sum of the contacts contributing to the Hirhsfeld surface of compound (**II**). The H⋯H inter­actions appear in the middle of the scattered points in the two-dimensional fingerprint plots, with a contribution to the overall Hirshfeld surface of 34.4% (Fig. 7 ▸ *b*). The contributions (16.5%) from the O⋯H/H⋯O contacts, corresponding to the C—H⋯O inter­actions, are represented by a pair of sharp spikes characteristic of such hydrogen bonding (Fig. 7 ▸ *d*).

## Synthesis and crystallization   

For the preparation of compound (**I**), sodium hydroxide (0.5 g, 3.5 mmol) was added to a solution (0.15 g, 1 mmol) of 6-phenyl-4,5-di­hydro­pyridazin-3(2*H*)-one and benzaldehyde (0.11 g, 1 mmol) in 30 ml of ethanol. The solvent was evaporated under vacuum and the residue was purified by silica-gel column chromatography using hexa­ne/ethyl acetate (7:3 *v*/*v*). Colourless single crystals were obtained by slow evaporation at room temperature.

For the preparation of compound (**II**), potassium carbonate (0.50 g, 3.5 mmol) was added to a solution (0.83 g, 2.5 mmol) of 4-(2,6-di­chloro­benz­yl)-6-phenyl­pyridazin-3(2*H*)-one in 30 ml of tetra­hydro­furan (THF). The mixture was refluxed for 1 h. After cooling, ethyl bromo­acetate (0.50 g, 3 mmol) was added and the mixture was refluxed for 8 h. The solid material which formed was removed by filtration and the solvent evaporated *in vacuo*. The residue was purified by silica-gel column chromatography using hexa­ne/ethyl acetate (4:6 *v*/*v*). Slow evaporation at room temperature led to colourless single crystals.

## Refinement   

Crystal data, data collection and structure refinement details are summarized in Table 3 ▸. For the structure of compound (**I**), the N-bound H atom was located in a difference Fourier map and refined with N—H = 0.86 Å. For the refinement of structure (**II**), reflections with a θ angle greater than 28° were omitted from the refinement due to their very weak intensities. The meth­oxy group (O3—C20) in this compound was found to be disordered over two sets of sites and was refined with an occupancy ratio of 0.626 (11):0.374 (11) (SIMU, DELU and ISOR commands in *SHELX*; Sheldrick, 2015*b* ▸). For both structures, the C-bound H atoms were positioned geometrically and refined using a riding model, with C—H = 0.93–0.97 Å and *U*_iso_(H) = 1.5*U*_eq_(C) for methyl H atoms or 1.2*U*_eq_(C) otherwise.

## Supplementary Material

Crystal structure: contains datablock(s) I, II, global. DOI: 10.1107/S2056989019013707/wm5515sup1.cif

Structure factors: contains datablock(s) I. DOI: 10.1107/S2056989019013707/wm5515Isup4.hkl

Structure factors: contains datablock(s) II. DOI: 10.1107/S2056989019013707/wm5515IIsup5.hkl

Supporting information file. DOI: 10.1107/S2056989019013707/wm5515Isup4.cml

Supporting information file. DOI: 10.1107/S2056989019013707/wm5515IIsup5.cml

CCDC references: 1958029, 1958030, 1958029, 1958030

Additional supporting information:  crystallographic information; 3D view; checkCIF report

## Figures and Tables

**Table 1:** Hydrogen-bond geometry (Å, °) for (**I**)

*D*—H⋯*A*	*D*—H	H⋯*A*	*D*⋯*A*	*D*—H⋯*A*
C10—H10⋯O1^i^	0.93	2.48	3.404 (2)	172
N1—H1⋯O1^ii^	0.937 (18)	1.855 (19)	2.7873 (19)	173.0 (16)

Symmetry codes: (i) 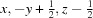; (ii) 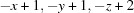.

**Table 2:** Hydrogen-bond geometry (Å, °) for (**II**)

*D*—H⋯*A*	*D*—H	H⋯*A*	*D*⋯*A*	*D*—H⋯*A*
C9—H9⋯O2^i^	0.93	2.50	3.337 (4)	150
C12—H12⋯O2^i^	0.93	2.40	3.326 (4)	174

Symmetry code: (i) 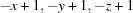.

**Table 3:** Experimental details

	(I)	(II)
Crystal data		
Chemical formula	C_17_H_14_N_2_O	C_20_H_16_Cl_2_N_2_O_3_
*M*_r_	262.30	403.25
Crystal system, space group	Monoclinic, *P*2_1_/*c*	Monoclinic, *P*2_1_/*c*
Temperature (K)	296	296
*a*, *b*, *c* (Å)	10.819 (3), 11.501 (3), 11.187 (4)	11.2730 (13), 12.3808 (9), 14.1405 (15)
β (°)	90.93 (3)	92.801 (9)
*V* (Å^3^)	1391.7 (7)	1971.2 (3)
*Z*	4	4
Radiation type	Mo *K*α	Mo *K*α
μ (mm^−1^)	0.08	0.35
Crystal size (mm)	0.78 × 0.71 × 0.59	0.80 × 0.76 × 0.60

Data collection		
Diffractometer	Stoe IPDS 2	Stoe IPDS 2
Absorption correction	Integration (*X-RED32*; Stoe & Cie, 2002 ▸)	Integration (*X-RED32*; Stoe & Cie, 2002 ▸)
*T*_min_, *T*_max_	0.943, 0.963	0.762, 0.832
No. of measured, independent and observed [*I* > 2σ(*I*)] reflections	14296, 4234, 1728	19293, 6015, 1892
*R*_int_	0.084	0.095
(sin θ/λ)_max_ (Å^−1^)	0.723	0.716

Refinement		
*R*[*F* ^2^ > 2σ(*F* ^2^)], *wR*(*F* ^2^), *S*	0.056, 0.104, 0.88	0.068, 0.231, 0.83
No. of reflections	4234	6015
No. of parameters	185	265
No. of restraints	0	68
H-atom treatment	H atoms treated by a mixture of independent and constrained refinement	H-atom parameters constrained
Δρ_max_, Δρ_min_ (e Å^−3^)	0.12, −0.22	0.96, −0.28

Computer programs: *X-AREA* (Stoe & Cie, 2002 ▸), *X-RED* (Stoe & Cie, 2002 ▸), *SHELXT2017* (Sheldrick, 2015*a* ▸), *SHELXL2017* (Sheldrick, 2015*b* ▸), *PLATON* (Spek, 2009 ▸) and *WinGX* (Farrugia, 2012 ▸).

## supplementary crystallographic information

### 4-Benzyl-6-phenyl-4,5-dihydropyridazin-3(2H)-one (I) Crystal data

**Table:**

C_17_H_14_N_2_O	*F*(000) = 552
*M**_r_* = 262.30	*D*_x_ = 1.252 Mg m^−^^3^
Monoclinic, *P*2_1_/*c*	Mo *K*α radiation, λ = 0.71073 Å
*a* = 10.819 (3) Å	Cell parameters from 15196 reflections
*b* = 11.501 (3) Å	θ = 3.2–31.3°
*c* = 11.187 (4) Å	µ = 0.08 mm^−^^1^
β = 90.93 (3)°	*T* = 296 K
*V* = 1391.7 (7) Å^3^	Prism, colourless
*Z* = 4	0.78 × 0.71 × 0.59 mm

### 4-Benzyl-6-phenyl-4,5-dihydropyridazin-3(2H)-one (I) Data collection

**Table:**

STOE IPDS 2 diffractometer	4234 independent reflections
Radiation source: sealed X-ray tube, 12 x 0.4 mm long-fine focus	1728 reflections with *I* > 2σ(*I*)
Detector resolution: 6.67 pixels mm^-1^	*R*_int_ = 0.084
rotation method scans	θ_max_ = 30.9°, θ_min_ = 3.2°
Absorption correction: integration (*X-RED32*; Stoe & Cie, 2002)	*h* = −13→15
*T*_min_ = 0.943, *T*_max_ = 0.963	*k* = −16→16
14296 measured reflections	*l* = −15→16

### 4-Benzyl-6-phenyl-4,5-dihydropyridazin-3(2H)-one (I) Refinement

**Table:**

Refinement on *F*^2^	0 restraints
Least-squares matrix: full	Hydrogen site location: mixed
*R*[*F*^2^ > 2σ(*F*^2^)] = 0.056	H atoms treated by a mixture of independent and constrained refinement
*wR*(*F*^2^) = 0.104	*w* = 1/[σ^2^(*F*_o_^2^) + (0.034*P*)^2^] where *P* = (*F*_o_^2^ + 2*F*_c_^2^)/3
*S* = 0.88	(Δ/σ)_max_ < 0.001
4234 reflections	Δρ_max_ = 0.12 e Å^−^^3^
185 parameters	Δρ_min_ = −0.22 e Å^−^^3^

### 4-Benzyl-6-phenyl-4,5-dihydropyridazin-3(2H)-one (I) Special details

**Table:**

Geometry. All esds (except the esd in the dihedral angle between two l.s. planes) are estimated using the full covariance matrix. The cell esds are taken into account individually in the estimation of esds in distances, angles and torsion angles; correlations between esds in cell parameters are only used when they are defined by crystal symmetry. An approximate (isotropic) treatment of cell esds is used for estimating esds involving l.s. planes.

### 4-Benzyl-6-phenyl-4,5-dihydropyridazin-3(2H)-one (I) Fractional atomic coordinates and isotropic or equivalent isotropic displacement parameters (Å^2^)

**Table:**

	*x*	*y*	*z*	*U*_iso_*/*U*_eq_	
O1	0.62706 (11)	0.40510 (10)	0.98172 (10)	0.0511 (3)	
N1	0.48091 (14)	0.45541 (13)	0.84500 (12)	0.0441 (4)	
N2	0.41272 (13)	0.44540 (12)	0.74360 (12)	0.0441 (4)	
C11	0.44527 (16)	0.36178 (14)	0.67190 (15)	0.0405 (4)	
C2	0.61718 (15)	0.30051 (13)	0.79846 (14)	0.0392 (4)	
C1	0.57821 (16)	0.38841 (14)	0.88184 (15)	0.0406 (4)	
C10	0.54855 (16)	0.28797 (14)	0.69831 (14)	0.0446 (4)	
H10	0.568712	0.229595	0.644512	0.053*	
C3	0.73380 (16)	0.23298 (14)	0.82536 (15)	0.0475 (4)	
H3A	0.728334	0.156834	0.788363	0.057*	
H3B	0.742972	0.222204	0.911046	0.057*	
C12	0.37007 (16)	0.34746 (15)	0.56177 (15)	0.0440 (4)	
C4	0.84563 (17)	0.29722 (15)	0.77837 (16)	0.0475 (4)	
C13	0.33000 (17)	0.23823 (18)	0.52768 (17)	0.0556 (5)	
H13	0.352747	0.173742	0.573115	0.067*	
C5	0.9009 (2)	0.38546 (17)	0.84222 (19)	0.0628 (5)	
H5	0.872935	0.403399	0.918191	0.075*	
C17	0.33608 (18)	0.44182 (17)	0.49299 (16)	0.0559 (5)	
H17	0.362199	0.515999	0.514775	0.067*	
C15	0.22392 (19)	0.3182 (2)	0.35938 (19)	0.0733 (6)	
H15	0.174705	0.308662	0.291153	0.088*	
C14	0.25661 (19)	0.2239 (2)	0.4269 (2)	0.0684 (6)	
H14	0.229389	0.150109	0.404994	0.082*	
C9	0.8903 (2)	0.2735 (2)	0.66590 (19)	0.0735 (6)	
H9	0.854388	0.214096	0.620805	0.088*	
C16	0.2635 (2)	0.4265 (2)	0.39206 (18)	0.0722 (6)	
H16	0.241251	0.490523	0.345696	0.087*	
C6	0.9983 (2)	0.4484 (2)	0.7947 (3)	0.0943 (9)	
H6	1.034628	0.507883	0.839257	0.113*	
C7	1.0412 (3)	0.4237 (3)	0.6833 (4)	0.1156 (12)	
H7	1.106222	0.465952	0.651715	0.139*	
C8	0.9869 (3)	0.3360 (3)	0.6192 (3)	0.1073 (10)	
H8	1.015410	0.318269	0.543388	0.129*	
H1	0.4486 (16)	0.5073 (16)	0.9010 (16)	0.060 (6)*	

### 4-Benzyl-6-phenyl-4,5-dihydropyridazin-3(2H)-one (I) Atomic displacement parameters (Å^2^)

**Table:**

	*U*^11^	*U*^22^	*U*^33^	*U*^12^	*U*^13^	*U*^23^
O1	0.0612 (8)	0.0586 (8)	0.0334 (6)	0.0067 (6)	−0.0021 (6)	−0.0081 (6)
N1	0.0550 (10)	0.0423 (9)	0.0351 (8)	0.0067 (7)	0.0024 (7)	−0.0069 (7)
N2	0.0504 (9)	0.0452 (9)	0.0366 (8)	0.0039 (7)	0.0012 (7)	−0.0033 (7)
C11	0.0447 (10)	0.0403 (10)	0.0367 (9)	−0.0009 (8)	0.0043 (8)	−0.0012 (7)
C2	0.0466 (10)	0.0351 (9)	0.0360 (9)	−0.0009 (8)	0.0024 (8)	0.0002 (7)
C1	0.0489 (11)	0.0405 (10)	0.0327 (10)	−0.0038 (9)	0.0053 (9)	0.0002 (7)
C10	0.0539 (11)	0.0399 (10)	0.0399 (10)	0.0019 (9)	0.0004 (9)	−0.0092 (8)
C3	0.0597 (12)	0.0403 (9)	0.0423 (9)	0.0075 (9)	−0.0055 (9)	−0.0020 (8)
C12	0.0397 (10)	0.0539 (11)	0.0384 (10)	0.0057 (9)	0.0028 (8)	−0.0086 (8)
C4	0.0434 (10)	0.0516 (10)	0.0474 (10)	0.0131 (9)	−0.0041 (9)	0.0031 (9)
C13	0.0514 (11)	0.0595 (12)	0.0556 (11)	0.0084 (10)	−0.0070 (10)	−0.0142 (10)
C5	0.0614 (13)	0.0666 (13)	0.0601 (13)	−0.0035 (11)	−0.0142 (11)	0.0065 (10)
C17	0.0572 (13)	0.0602 (12)	0.0501 (11)	0.0030 (10)	−0.0025 (10)	0.0021 (10)
C15	0.0566 (14)	0.113 (2)	0.0494 (12)	0.0119 (14)	−0.0123 (10)	−0.0194 (14)
C14	0.0531 (13)	0.0787 (15)	0.0731 (14)	0.0052 (11)	−0.0094 (11)	−0.0305 (13)
C9	0.0715 (15)	0.0874 (16)	0.0619 (13)	0.0268 (13)	0.0118 (12)	−0.0014 (12)
C16	0.0694 (15)	0.0965 (18)	0.0504 (12)	0.0146 (13)	−0.0086 (12)	0.0098 (12)
C6	0.0636 (16)	0.097 (2)	0.121 (2)	−0.0194 (15)	−0.0214 (17)	0.0302 (18)
C7	0.0527 (17)	0.144 (3)	0.151 (3)	0.0057 (18)	0.0204 (19)	0.063 (3)
C8	0.077 (2)	0.149 (3)	0.096 (2)	0.036 (2)	0.0412 (17)	0.022 (2)

### 4-Benzyl-6-phenyl-4,5-dihydropyridazin-3(2H)-one (I) Geometric parameters (Å, º)

**Table:**

O1—C1	1.243 (2)	C13—C14	1.378 (3)
N1—N2	1.348 (2)	C13—H13	0.9300
N1—C1	1.363 (2)	C5—C6	1.390 (3)
N1—H1	0.937 (18)	C5—H5	0.9300
N2—C11	1.3044 (19)	C17—C16	1.376 (3)
C11—C10	1.430 (2)	C17—H17	0.9300
C11—C12	1.475 (2)	C15—C14	1.365 (3)
C2—C10	1.342 (2)	C15—C16	1.365 (3)
C2—C1	1.443 (2)	C15—H15	0.9300
C2—C3	1.508 (2)	C14—H14	0.9300
C10—H10	0.9300	C9—C8	1.379 (4)
C3—C4	1.519 (2)	C9—H9	0.9300
C3—H3A	0.9700	C16—H16	0.9300
C3—H3B	0.9700	C6—C7	1.367 (4)
C12—C17	1.377 (2)	C6—H6	0.9300
C12—C13	1.381 (2)	C7—C8	1.364 (4)
C4—C5	1.373 (3)	C7—H7	0.9300
C4—C9	1.382 (3)	C8—H8	0.9300
			
N2—N1—C1	128.00 (14)	C14—C13—H13	119.7
N2—N1—H1	114.4 (11)	C12—C13—H13	119.7
C1—N1—H1	116.9 (11)	C4—C5—C6	120.8 (2)
C11—N2—N1	115.52 (15)	C4—C5—H5	119.6
N2—C11—C10	121.89 (16)	C6—C5—H5	119.6
N2—C11—C12	116.44 (15)	C16—C17—C12	120.1 (2)
C10—C11—C12	121.67 (15)	C16—C17—H17	120.0
C10—C2—C1	116.83 (16)	C12—C17—H17	120.0
C10—C2—C3	124.15 (15)	C14—C15—C16	120.0 (2)
C1—C2—C3	118.96 (15)	C14—C15—H15	120.0
O1—C1—N1	119.96 (15)	C16—C15—H15	120.0
O1—C1—C2	124.38 (17)	C15—C14—C13	119.9 (2)
N1—C1—C2	115.66 (15)	C15—C14—H14	120.1
C2—C10—C11	121.92 (15)	C13—C14—H14	120.1
C2—C10—H10	119.0	C8—C9—C4	121.3 (2)
C11—C10—H10	119.0	C8—C9—H9	119.3
C2—C3—C4	110.44 (14)	C4—C9—H9	119.3
C2—C3—H3A	109.6	C15—C16—C17	120.6 (2)
C4—C3—H3A	109.6	C15—C16—H16	119.7
C2—C3—H3B	109.6	C17—C16—H16	119.7
C4—C3—H3B	109.6	C7—C6—C5	120.7 (3)
H3A—C3—H3B	108.1	C7—C6—H6	119.6
C17—C12—C13	118.88 (17)	C5—C6—H6	119.6
C17—C12—C11	121.17 (16)	C8—C7—C6	118.9 (3)
C13—C12—C11	119.93 (17)	C8—C7—H7	120.5
C5—C4—C9	117.6 (2)	C6—C7—H7	120.5
C5—C4—C3	121.50 (17)	C7—C8—C9	120.6 (3)
C9—C4—C3	120.73 (19)	C7—C8—H8	119.7
C14—C13—C12	120.6 (2)	C9—C8—H8	119.7
			
C1—N1—N2—C11	−0.7 (2)	C2—C3—C4—C5	82.6 (2)
N1—N2—C11—C10	−1.6 (2)	C2—C3—C4—C9	−93.2 (2)
N1—N2—C11—C12	178.58 (15)	C17—C12—C13—C14	−0.4 (3)
N2—N1—C1—O1	−175.85 (16)	C11—C12—C13—C14	178.07 (17)
N2—N1—C1—C2	4.0 (2)	C9—C4—C5—C6	0.1 (3)
C10—C2—C1—O1	174.96 (17)	C3—C4—C5—C6	−175.88 (18)
C3—C2—C1—O1	−7.8 (2)	C13—C12—C17—C16	−0.1 (3)
C10—C2—C1—N1	−4.9 (2)	C11—C12—C17—C16	−178.52 (17)
C3—C2—C1—N1	172.33 (14)	C16—C15—C14—C13	−0.3 (3)
C1—C2—C10—C11	3.1 (2)	C12—C13—C14—C15	0.6 (3)
C3—C2—C10—C11	−173.96 (15)	C5—C4—C9—C8	0.0 (3)
N2—C11—C10—C2	0.3 (3)	C3—C4—C9—C8	176.0 (2)
C12—C11—C10—C2	−179.90 (16)	C14—C15—C16—C17	−0.2 (3)
C10—C2—C3—C4	90.0 (2)	C12—C17—C16—C15	0.4 (3)
C1—C2—C3—C4	−86.97 (18)	C4—C5—C6—C7	0.0 (4)
N2—C11—C12—C17	45.2 (2)	C5—C6—C7—C8	−0.1 (4)
C10—C11—C12—C17	−134.69 (17)	C6—C7—C8—C9	0.1 (4)
N2—C11—C12—C13	−133.26 (17)	C4—C9—C8—C7	−0.1 (4)
C10—C11—C12—C13	46.9 (2)		

### 4-Benzyl-6-phenyl-4,5-dihydropyridazin-3(2H)-one (I) Hydrogen-bond geometry (Å, º)

**Table:**

*D*—H···*A*	*D*—H	H···*A*	*D*···*A*	*D*—H···*A*
C10—H10···O1^i^	0.93	2.48	3.404 (2)	172
N1—H1···O1^ii^	0.937 (18)	1.855 (19)	2.7873 (19)	173.0 (16)

Symmetry codes: (i) *x*, −*y*+1/2, *z*−1/2; (ii) −*x*+1, −*y*+1, −*z*+2.

### Methyl 2-[5-(2,6-dichlorobenzyl)-6-oxo-3-phenyl-1,4,5,6-tetrahydro-pyridazin-1-yl]acetate (II) Crystal data

**Table:**

C_20_H_16_Cl_2_N_2_O_3_	*F*(000) = 832
*M**_r_* = 403.25	*D*_x_ = 1.359 Mg m^−^^3^
Monoclinic, *P*2_1_/*c*	Mo *K*α radiation, λ = 0.71073 Å
*a* = 11.2730 (13) Å	Cell parameters from 14081 reflections
*b* = 12.3808 (9) Å	θ = 1.6–30.5°
*c* = 14.1405 (15) Å	µ = 0.35 mm^−^^1^
β = 92.801 (9)°	*T* = 296 K
*V* = 1971.2 (3) Å^3^	Cubic, colourless
*Z* = 4	0.80 × 0.76 × 0.60 mm

### Methyl 2-[5-(2,6-dichlorobenzyl)-6-oxo-3-phenyl-1,4,5,6-tetrahydro-pyridazin-1-yl]acetate (II) Data collection

**Table:**

STOE IPDS 2 diffractometer	6015 independent reflections
Radiation source: sealed X-ray tube, 12 x 0.4 mm long-fine focus	1892 reflections with *I* > 2σ(*I*)
Detector resolution: 6.67 pixels mm^-1^	*R*_int_ = 0.095
rotation method scans	θ_max_ = 30.6°, θ_min_ = 1.8°
Absorption correction: integration (*X-RED32*; Stoe & Cie, 2002)	*h* = −14→16
*T*_min_ = 0.762, *T*_max_ = 0.832	*k* = −17→17
19293 measured reflections	*l* = −20→20

### Methyl 2-[5-(2,6-dichlorobenzyl)-6-oxo-3-phenyl-1,4,5,6-tetrahydro-pyridazin-1-yl]acetate (II) Refinement

**Table:**

Refinement on *F*^2^	68 restraints
Least-squares matrix: full	Hydrogen site location: inferred from neighbouring sites
*R*[*F*^2^ > 2σ(*F*^2^)] = 0.068	H-atom parameters constrained
*wR*(*F*^2^) = 0.231	*w* = 1/[σ^2^(*F*_o_^2^) + (0.1133*P*)^2^] where *P* = (*F*_o_^2^ + 2*F*_c_^2^)/3
*S* = 0.83	(Δ/σ)_max_ < 0.001
6015 reflections	Δρ_max_ = 0.96 e Å^−^^3^
265 parameters	Δρ_min_ = −0.28 e Å^−^^3^

### Methyl 2-[5-(2,6-dichlorobenzyl)-6-oxo-3-phenyl-1,4,5,6-tetrahydro-pyridazin-1-yl]acetate (II) Special details

**Table:**

Geometry. All esds (except the esd in the dihedral angle between two l.s. planes) are estimated using the full covariance matrix. The cell esds are taken into account individually in the estimation of esds in distances, angles and torsion angles; correlations between esds in cell parameters are only used when they are defined by crystal symmetry. An approximate (isotropic) treatment of cell esds is used for estimating esds involving l.s. planes.

### Methyl 2-[5-(2,6-dichlorobenzyl)-6-oxo-3-phenyl-1,4,5,6-tetrahydro-pyridazin-1-yl]acetate (II) Fractional atomic coordinates and isotropic or equivalent isotropic displacement parameters (Å^2^)

**Table:**

	*x*	*y*	*z*	*U*_iso_*/*U*_eq_	Occ. (<1)
Cl2	0.28302 (11)	0.78380 (8)	0.39545 (9)	0.0874 (4)	
Cl1	0.11530 (12)	0.56182 (11)	0.68990 (9)	0.0990 (4)	
O1	0.4761 (2)	0.6024 (2)	0.73816 (19)	0.0734 (8)	
O2	0.7312 (3)	0.4765 (2)	0.6889 (2)	0.0785 (8)	
N2	0.4886 (3)	0.4295 (2)	0.6878 (2)	0.0598 (8)	
N1	0.4620 (3)	0.3458 (2)	0.6290 (2)	0.0589 (8)	
C10	0.3921 (3)	0.3643 (2)	0.5550 (2)	0.0521 (8)	
C9	0.3431 (3)	0.4694 (2)	0.5375 (3)	0.0525 (8)	
H9	0.293991	0.480718	0.483617	0.063*	
C8	0.3664 (3)	0.5517 (2)	0.5968 (2)	0.0522 (8)	
C6	0.1935 (3)	0.6695 (3)	0.5385 (3)	0.0575 (9)	
C17	0.4456 (3)	0.5342 (3)	0.6787 (3)	0.0570 (9)	
C11	0.3627 (3)	0.2690 (3)	0.4945 (3)	0.0555 (9)	
C5	0.1694 (3)	0.7216 (3)	0.4534 (3)	0.0645 (10)	
C7	0.3150 (3)	0.6633 (3)	0.5872 (3)	0.0624 (10)	
H7A	0.368806	0.707534	0.552254	0.075*	
H7B	0.311027	0.694349	0.649953	0.075*	
C19	0.6926 (4)	0.4357 (3)	0.7570 (3)	0.0660 (9)	
C12	0.3198 (3)	0.2793 (3)	0.4022 (3)	0.0636 (10)	
H12	0.311970	0.347892	0.375777	0.076*	
C18	0.5661 (3)	0.4012 (3)	0.7686 (3)	0.0659 (10)	
H18A	0.563881	0.323646	0.777873	0.079*	
H18B	0.537030	0.435149	0.824835	0.079*	
C1	0.0960 (4)	0.6252 (3)	0.5811 (3)	0.0689 (10)	
C16	0.3768 (4)	0.1655 (3)	0.5324 (3)	0.0702 (11)	
H16	0.406443	0.156363	0.594423	0.084*	
C13	0.2881 (4)	0.1900 (3)	0.3477 (3)	0.0761 (12)	
H13	0.258183	0.199013	0.285767	0.091*	
C14	0.3009 (4)	0.0890 (3)	0.3850 (3)	0.0779 (12)	
H14	0.279112	0.028804	0.348834	0.093*	
C15	0.3463 (4)	0.0763 (3)	0.4771 (3)	0.0832 (13)	
H15	0.356513	0.007258	0.502085	0.100*	
C4	0.0557 (4)	0.7290 (4)	0.4124 (4)	0.0859 (13)	
H4	0.042696	0.765185	0.355204	0.103*	
C2	−0.0182 (4)	0.6326 (4)	0.5418 (4)	0.0892 (14)	
H2	−0.081667	0.603691	0.572954	0.107*	
C3	−0.0371 (4)	0.6835 (4)	0.4557 (4)	0.0965 (16)	
H3	−0.113140	0.686665	0.427209	0.116*	
O3A	0.7602 (3)	0.3969 (6)	0.8303 (3)	0.0728 (15)	0.626 (11)
C20A	0.8977 (5)	0.4168 (8)	0.8327 (6)	0.092 (2)	0.626 (11)
H20A	0.918846	0.449346	0.774225	0.138*	0.626 (11)
H20B	0.938559	0.349210	0.841005	0.138*	0.626 (11)
H20C	0.919935	0.464147	0.884368	0.138*	0.626 (11)
O3B	0.7492 (6)	0.4387 (10)	0.8431 (4)	0.0755 (19)	0.374 (11)
C20B	0.8759 (9)	0.4925 (14)	0.8535 (8)	0.091 (3)	0.374 (11)
H20D	0.916704	0.482091	0.796241	0.137*	0.374 (11)
H20E	0.920606	0.459831	0.905487	0.137*	0.374 (11)
H20F	0.867405	0.568435	0.865276	0.137*	0.374 (11)

### Methyl 2-[5-(2,6-dichlorobenzyl)-6-oxo-3-phenyl-1,4,5,6-tetrahydro-pyridazin-1-yl]acetate (II) Atomic displacement parameters (Å^2^)

**Table:**

	*U*^11^	*U*^22^	*U*^33^	*U*^12^	*U*^13^	*U*^23^
Cl2	0.1012 (9)	0.0618 (6)	0.0996 (9)	0.0013 (6)	0.0092 (7)	0.0122 (6)
Cl1	0.1100 (10)	0.1085 (9)	0.0791 (8)	−0.0063 (7)	0.0106 (7)	0.0075 (7)
O1	0.0876 (19)	0.0628 (15)	0.0671 (17)	−0.0012 (13)	−0.0224 (14)	−0.0133 (13)
O2	0.0850 (19)	0.0732 (17)	0.0761 (17)	−0.0032 (14)	−0.0089 (15)	0.0184 (15)
N2	0.0670 (19)	0.0536 (17)	0.0570 (17)	0.0014 (14)	−0.0160 (15)	0.0007 (15)
N1	0.0655 (19)	0.0484 (16)	0.0619 (18)	0.0012 (14)	−0.0076 (15)	0.0044 (15)
C10	0.0540 (19)	0.0444 (17)	0.057 (2)	−0.0001 (14)	−0.0094 (16)	0.0043 (16)
C9	0.0537 (19)	0.0460 (17)	0.056 (2)	0.0014 (14)	−0.0114 (16)	0.0009 (15)
C8	0.0517 (19)	0.0446 (17)	0.059 (2)	−0.0005 (14)	−0.0086 (15)	−0.0009 (15)
C6	0.061 (2)	0.0389 (17)	0.071 (2)	0.0010 (15)	−0.0111 (18)	−0.0091 (17)
C17	0.060 (2)	0.0491 (19)	0.061 (2)	0.0023 (16)	−0.0075 (17)	−0.0020 (17)
C11	0.060 (2)	0.0422 (18)	0.064 (2)	0.0032 (15)	−0.0006 (17)	−0.0027 (16)
C5	0.070 (2)	0.0454 (18)	0.078 (3)	0.0046 (17)	0.000 (2)	−0.0051 (18)
C7	0.063 (2)	0.0433 (18)	0.079 (3)	0.0013 (16)	−0.0172 (19)	−0.0063 (18)
C19	0.079 (2)	0.0544 (19)	0.062 (2)	−0.0015 (17)	−0.0175 (17)	0.0069 (17)
C12	0.079 (2)	0.0496 (19)	0.062 (2)	0.0016 (18)	−0.0031 (19)	−0.0021 (17)
C18	0.067 (2)	0.068 (2)	0.061 (2)	0.0009 (19)	−0.0169 (19)	0.0138 (19)
C1	0.068 (3)	0.059 (2)	0.079 (3)	0.0023 (18)	−0.005 (2)	−0.0094 (19)
C16	0.093 (3)	0.048 (2)	0.068 (2)	0.0014 (19)	−0.012 (2)	0.0026 (19)
C13	0.097 (3)	0.066 (3)	0.064 (2)	0.004 (2)	−0.005 (2)	−0.013 (2)
C14	0.097 (3)	0.054 (2)	0.082 (3)	−0.003 (2)	−0.001 (2)	−0.017 (2)
C15	0.115 (4)	0.044 (2)	0.090 (3)	−0.003 (2)	−0.001 (3)	−0.004 (2)
C4	0.087 (3)	0.079 (3)	0.089 (3)	0.017 (2)	−0.022 (3)	0.004 (2)
C2	0.063 (3)	0.093 (3)	0.111 (4)	0.000 (2)	0.000 (3)	−0.010 (3)
C3	0.060 (3)	0.105 (4)	0.122 (4)	0.011 (3)	−0.025 (3)	−0.009 (3)
O3A	0.075 (2)	0.068 (3)	0.072 (2)	−0.011 (2)	−0.025 (2)	0.011 (2)
C20A	0.087 (3)	0.083 (5)	0.105 (5)	−0.025 (4)	−0.008 (4)	0.018 (4)
O3B	0.080 (3)	0.074 (4)	0.070 (3)	−0.009 (3)	−0.023 (3)	0.005 (3)
C20B	0.072 (5)	0.102 (6)	0.100 (6)	0.003 (5)	0.001 (4)	0.024 (6)

### Methyl 2-[5-(2,6-dichlorobenzyl)-6-oxo-3-phenyl-1,4,5,6-tetrahydro-pyridazin-1-yl]acetate (II) Geometric parameters (Å, º)

**Table:**

Cl2—C5	1.734 (4)	C12—C13	1.384 (5)
Cl1—C1	1.731 (4)	C12—H12	0.9300
O1—C17	1.228 (4)	C18—H18A	0.9700
O2—C19	1.189 (5)	C18—H18B	0.9700
N2—N1	1.353 (4)	C1—C2	1.380 (6)
N2—C17	1.388 (4)	C16—C15	1.387 (5)
N2—C18	1.447 (4)	C16—H16	0.9300
N1—C10	1.299 (4)	C13—C14	1.363 (6)
C10—C9	1.430 (4)	C13—H13	0.9300
C10—C11	1.484 (4)	C14—C15	1.384 (6)
C9—C8	1.338 (4)	C14—H14	0.9300
C9—H9	0.9300	C15—H15	0.9300
C8—C17	1.444 (5)	C4—C3	1.359 (7)
C8—C7	1.503 (4)	C4—H4	0.9300
C6—C5	1.381 (5)	C2—C3	1.378 (7)
C6—C1	1.392 (6)	C2—H2	0.9300
C6—C7	1.504 (5)	C3—H3	0.9300
C11—C12	1.376 (5)	O3A—C20A	1.569 (6)
C11—C16	1.395 (5)	C20A—H20A	0.9600
C5—C4	1.384 (6)	C20A—H20B	0.9600
C7—H7A	0.9700	C20A—H20C	0.9600
C7—H7B	0.9700	O3B—C20B	1.576 (7)
C19—O3A	1.344 (5)	C20B—H20D	0.9600
C19—O3B	1.347 (5)	C20B—H20E	0.9600
C19—C18	1.505 (6)	C20B—H20F	0.9600
			
N1—N2—C17	126.3 (3)	N2—C18—H18B	109.1
N1—N2—C18	114.1 (3)	C19—C18—H18B	109.1
C17—N2—C18	119.6 (3)	H18A—C18—H18B	107.8
C10—N1—N2	117.9 (3)	C2—C1—C6	122.7 (4)
N1—C10—C9	120.9 (3)	C2—C1—Cl1	117.6 (4)
N1—C10—C11	115.7 (3)	C6—C1—Cl1	119.7 (3)
C9—C10—C11	123.3 (3)	C15—C16—C11	119.6 (4)
C8—C9—C10	121.5 (3)	C15—C16—H16	120.2
C8—C9—H9	119.3	C11—C16—H16	120.2
C10—C9—H9	119.3	C14—C13—C12	119.9 (4)
C9—C8—C17	118.9 (3)	C14—C13—H13	120.1
C9—C8—C7	125.6 (3)	C12—C13—H13	120.1
C17—C8—C7	115.5 (3)	C13—C14—C15	119.7 (4)
C5—C6—C1	115.8 (3)	C13—C14—H14	120.2
C5—C6—C7	123.9 (4)	C15—C14—H14	120.2
C1—C6—C7	120.2 (3)	C14—C15—C16	120.7 (4)
O1—C17—N2	119.6 (3)	C14—C15—H15	119.7
O1—C17—C8	126.0 (3)	C16—C15—H15	119.7
N2—C17—C8	114.4 (3)	C3—C4—C5	120.1 (4)
C12—C11—C16	118.5 (3)	C3—C4—H4	120.0
C12—C11—C10	122.1 (3)	C5—C4—H4	120.0
C16—C11—C10	119.4 (3)	C3—C2—C1	119.2 (5)
C6—C5—C4	122.3 (4)	C3—C2—H2	120.4
C6—C5—Cl2	120.1 (3)	C1—C2—H2	120.4
C4—C5—Cl2	117.6 (4)	C4—C3—C2	119.9 (4)
C8—C7—C6	115.2 (3)	C4—C3—H3	120.1
C8—C7—H7A	108.5	C2—C3—H3	120.1
C6—C7—H7A	108.5	C19—O3A—C20A	118.9 (4)
C8—C7—H7B	108.5	O3A—C20A—H20A	109.5
C6—C7—H7B	108.5	O3A—C20A—H20B	109.5
H7A—C7—H7B	107.5	H20A—C20A—H20B	109.5
O2—C19—O3A	124.0 (4)	O3A—C20A—H20C	109.5
O2—C19—O3B	123.0 (4)	H20A—C20A—H20C	109.5
O2—C19—C18	126.5 (3)	H20B—C20A—H20C	109.5
O3A—C19—C18	108.6 (4)	C19—O3B—C20B	118.9 (5)
O3B—C19—C18	108.6 (4)	O3B—C20B—H20D	109.5
C11—C12—C13	121.6 (3)	O3B—C20B—H20E	109.5
C11—C12—H12	119.2	H20D—C20B—H20E	109.5
C13—C12—H12	119.2	O3B—C20B—H20F	109.5
N2—C18—C19	112.6 (3)	H20D—C20B—H20F	109.5
N2—C18—H18A	109.1	H20E—C20B—H20F	109.5
C19—C18—H18A	109.1		
			
C17—N2—N1—C10	1.9 (5)	C16—C11—C12—C13	1.6 (6)
C18—N2—N1—C10	179.5 (3)	C10—C11—C12—C13	−177.3 (4)
N2—N1—C10—C9	−1.2 (5)	N1—N2—C18—C19	102.0 (4)
N2—N1—C10—C11	−178.0 (3)	C17—N2—C18—C19	−80.2 (4)
N1—C10—C9—C8	−0.7 (6)	O2—C19—C18—N2	−4.0 (6)
C11—C10—C9—C8	176.0 (3)	O3A—C19—C18—N2	−173.8 (4)
C10—C9—C8—C17	1.8 (5)	O3B—C19—C18—N2	160.7 (6)
C10—C9—C8—C7	−176.9 (4)	C5—C6—C1—C2	−0.2 (6)
N1—N2—C17—O1	179.1 (3)	C7—C6—C1—C2	177.1 (4)
C18—N2—C17—O1	1.7 (5)	C5—C6—C1—Cl1	−177.9 (3)
N1—N2—C17—C8	−0.8 (5)	C7—C6—C1—Cl1	−0.6 (5)
C18—N2—C17—C8	−178.2 (3)	C12—C11—C16—C15	−0.7 (6)
C9—C8—C17—O1	179.0 (4)	C10—C11—C16—C15	178.2 (4)
C7—C8—C17—O1	−2.1 (6)	C11—C12—C13—C14	−1.0 (7)
C9—C8—C17—N2	−1.1 (5)	C12—C13—C14—C15	−0.5 (7)
C7—C8—C17—N2	177.8 (3)	C13—C14—C15—C16	1.3 (7)
N1—C10—C11—C12	−160.9 (3)	C11—C16—C15—C14	−0.7 (7)
C9—C10—C11—C12	22.3 (6)	C6—C5—C4—C3	−0.4 (7)
N1—C10—C11—C16	20.2 (5)	Cl2—C5—C4—C3	−178.8 (4)
C9—C10—C11—C16	−156.6 (4)	C6—C1—C2—C3	1.7 (7)
C1—C6—C5—C4	−0.4 (5)	Cl1—C1—C2—C3	179.4 (4)
C7—C6—C5—C4	−177.6 (4)	C5—C4—C3—C2	1.9 (7)
C1—C6—C5—Cl2	178.0 (3)	C1—C2—C3—C4	−2.5 (7)
C7—C6—C5—Cl2	0.8 (5)	O2—C19—O3A—C20A	6.9 (8)
C9—C8—C7—C6	29.2 (6)	C18—C19—O3A—C20A	177.1 (5)
C17—C8—C7—C6	−149.6 (3)	O2—C19—O3B—C20B	−3.6 (13)
C5—C6—C7—C8	−116.0 (4)	C18—C19—O3B—C20B	−168.9 (8)
C1—C6—C7—C8	66.9 (5)		

### Methyl 2-[5-(2,6-dichlorobenzyl)-6-oxo-3-phenyl-1,4,5,6-tetrahydro-pyridazin-1-yl]acetate (II) Hydrogen-bond geometry (Å, º)

**Table:**

*D*—H···*A*	*D*—H	H···*A*	*D*···*A*	*D*—H···*A*
C9—H9···O2^i^	0.93	2.50	3.337 (4)	150
C12—H12···O2^i^	0.93	2.40	3.326 (4)	174

Symmetry code: (i) −*x*+1, −*y*+1, −*z*+1.
